# The association between L:M cone ratio, cone opsin genes and myopia susceptibility

**DOI:** 10.1016/j.visres.2019.06.006

**Published:** 2019-07-11

**Authors:** Lene A. Hagen, Solveig Arnegard, James A. Kuchenbecker, Stuart J. Gilson, Maureen Neitz, Jay Neitz, Rigmor C. Baraas

**Affiliations:** a National Centre for Optics, Vision and Eye Care, Faculty of Health and Social Sciences, University of South-Eastern Norway, Hasbergs vei 36, 3616 Kongsberg, Norway; b Department of Ophthalmology, University of Washington Medical School, Box 358058, 750 Republican Street, Building E Room, Seattle, WA 98109, United States

**Keywords:** Myopia, Myopia susceptibility, Cone opsin genetics, L:M cone ratio, Color vision

## Abstract

In syndromic forms of myopia caused by long (L) to middle (M) wavelength (L/M) interchange mutations, erroneous contrast signals from ON-bipolar cells activated by cones with different levels of opsin expression are suggested to make the eye susceptible to increased growth. This susceptibility is modulated by the L:M cone ratio. Here, we examined L and M opsin genes, L:M cone ratios and their association with common refractive errors in a population with low myopia prevalence. Cycloplegic autorefraction and ocular biometry were obtained for Norwegian genetically-confirmed normal trichromats. L:M cone ratios were estimated from spectral sensitivity functions measured with full-field ERG, after adjusting for individual differences in the wavelength of peak absorption deduced from cone opsin genetics. Mean L:M cone ratios and the frequency of alanine at L opsin position 180 were higher in males than what has been reported in males in populations with high myopia prevalence. High L:M cone ratios in females were associated with lower degree of myopia, and myopia was more frequent in females who were heterozygous for L opsin exon 3 haplotypes than in those who were homozygous. The results suggest that the L:M cone ratio, combined with milder versions of L opsin gene polymorphisms, may play a role in common myopia. This may in part explain the low myopia prevalence in Norwegian adolescents and why myopia prevalence was higher in females who were heterozygous for the L opsin exon 3 haplotype, since females are twice as likely to have genetic polymorphisms carried on the X-chromosome.

## 1. Introduction

The prevalence of myopia is increasing around the world, including an associated increased risk of myopia-related complications (Holden et al., 2016). Ethnic and regional differences in myopia prevalence are reported, with East Asians (Pan, Ramamurthy, & Saw, 2012; Rudnicka et al., 2016) having a considerably higher prevalence than Caucasians (Hagen et al., 2018; McCullough, O’Donoghue, & Saunders, 2016). There is no general agreement on the etiology of myopia, but eye growth is primarily regulated by visual signals – processed locally – in the retina (Wallman & Winawer, 2004). The cone photoreceptors are the first step in the signalling cascade and, consequently, are likely to play a role in susceptibility to myopia development.

The human retina contains three classes of cone photoreceptor that are sensitive to light of long (L), middle (M) or short (S) wavelengths. The relative number of L and M cones (L:M cone ratio) varies between individuals, and the mean ratio differs between ethnic groups. A mean L:M cone ratio of 2.7:1 (~ 73% L cones) has been reported in colour normal American Caucasian males (Carroll, Neitz, & Neitz, 2002; Hofer, Carroll, Neitz, Neitz, & Williams, 2005). In East Asians with reported earlier myopia onset and higher myopia prevalence, the mean L:M cone ratio in colour normal males has been reported to be considerably lower than in American Caucasians (Kuchenbecker, Neitz, & Neitz, 2014; Yamauchi, Yatsu, Kuchenbecker, Neitz, & Neitz, 2013). Refraction and vitreous chamber depth are found to be associated with cone ratio in chickens (Gisbert & Schaeffel, 2018). In humans, an association between symmetric L:M cone ratios (near 1:1) and high susceptibility to myopia development has been proposed (Neitz & Neitz, 2015; Zhou, Atchison, Zele, Brown, & Schmid, 2015). There is evidence for this in the fact that there is a lower prevalence of myopia in red-green colour vision deficient students, who have highly skewed L:M cone ratios as a consequence of lacking L or M cones (Ostadimoghaddam et al., 2014; Qian et al., 2009). Further evidence is the association between myopia and rare interchange haplotypes in exon 3 of the L and M cone opsin genes at chromosome location Xq28 (designated OPN1LW and OPN1MW, respectively) (Carroll et al., 2012; Greenwald, Kuchenbecker, Rowlan, Neitz, & Neitz, 2017; McClements et al., 2013; Orosz et al., 2017). The highly variable nucleotide sequences in humans control the spectral tuning of the opsin and affect other aspects of protein structure and function, such as proper splicing of exon 3 in the precursor messenger RNA (pre-mRNA) (Greenwald et al., 2017). In syndromic forms of myopia caused by L/M interchange mutations, incorrect exon 3 splicing leads to greatly reduced amount of functional opsin – or no functional opsin at all – in the affected cones. In such a cone mosaic, neighbouring cones will have different levels of opsin expression. A normally functioning cone that is adjacent to a less-than-normally functioning cone will activate ON-bipolar cells even when there is no spatial contrast information in the visual scene/stimulus. Eye growth is, in these cases, suggested to be modulated by erroneous contrast signals produced by a mosaic of cones with different levels of opsin expression. The degree of erroneous signalling and myopia susceptibility depend on how many cones express the mutant opsin (Greenwald et al., 2017; Patterson et al., 2018). We are hypothesizing that it is not unlikely that other opsin gene exon 3 haplotypes with less severe splicing defects could play a role in common myopia (Neitz & Neitz, 2018). If so, heterozygosity of exon 3 haplotypes could increase myopia susceptibility in females resulting in earlier myopia onset (Rudnicka et al., 2016), because females are twice as likely to carry a cone opsin polymorphism on one of their two X-chromosomes. Heterozygosity of opsin haplotypes in females would translate into a retina where there will be patches with two sets of L and/or M cones expressing different haplotypes, and in the case of an exon 3 splicing defect, one set may give rise to less-than-normally functioning opsin.

If the L:M cone ratio and exon 3 haplotypes play a role in susceptibility to myopia (Greenwald et al., 2017; Neitz & Neitz, 2015; Zhou et al., 2015), it follows that L:M cone ratios, on average, may be higher in a population with low myopia prevalence. Furthermore, differences in exon 3 haplotypes may be observed between myopes and non-myopes, as would difference in myopia prevalence between heterozygous and homozygous females. The current study tested these hypotheses. Its aim, therefore, was to examine L and M opsin genes, L:M cone ratios and their association with refractive errors in Norwegian adolescent males and females. This is a population with low myopia prevalence, despite few daylight hours in the autumn-winter period, large amount of time spent indoors doing near work, and having one of the highest performing education systems in the world according to the Organisation for Economic Co-operation and Development (OECD) (Hagen et al., 2018). L:M cone ratios were estimated with full-field ERG flicker photometry. This is an efficient and reliable procedure for measuring L:M cone ratio when corrections are made for individual differences in the wavelength of peak absorption (λ_max_) of the L cone opsin and the optical density of the lens (Carroll, McMahon, Neitz, & Neitz, 2000; Carroll et al., 2002).

## 2. Methods

### 2. 1. Participants

One hundred and thirty-six genetically-confirmed normal trichromats [mean (± SD) age: 16.9 (± 1.0) yrs; 60 males] were included in the study. The participants were recruited by invitation, and the inclusion criteria were: Caucasian ethnicity, age 16–19 years, normal colour vision, being healthy with no known ocular abnormalities and no medication, stereo acuity ≤ 120” (TNO-test), and normal corrected visual acuity. This is a representative subsample of the participants who were included in a larger study of refractive errors in 16–19 year old Norwegian upper secondary school students [*n* = 393, 16.7 (± 0.9) yrs] (Hagen et al., 2018) in terms of sex [44.1% vs 41.2% males; χ^2^(1) = 0.24, *p* = 0.62] and proportion of refractive errors within the groups of males [8.3% vs 8.6% myopia; 58.3% vs 57.4% hyperopia; χ^2^(2) = 0.02, *p* = 0.99] and females [19.7% vs 15.6% myopia; 51.3% vs 56.3% hyperopia; χ^2^(2) = 0.86, *p* = 0.65]. All were Norwegian Caucasians living in Southeast Norway with normal habitual visual acuity both in the right and left eye [mean logMAR −0.01 (SD: 0.12; range: −0.26–0.62) and mean logMAR −0.02 (SD: 0.13; range: −0.24–0.54), respectively]. Huvitz HRK-8000A Auto-REF Keratometer (Huvitz Co. Ltd., Gyeonggi-do, Korea) and Zeiss IOLMaster (Carl Zeiss Meditec AG, Jena, Germany) were used to measure cycloplegic autorefraction and ocular biometry, described in detail elsewhere (Hagen et al., 2018). For validation of ERG measurements and estimates of L:M cone ratios, five red-green colour vision deficient males (13–66 yrs; 3 single gene dichromats) and one protan carrier (27 yrs) were included.

Colour vision status was confirmed in all participants by genetics, as well as by Ishihara (24 pl. ed., Kanehara Trading INC, Tokyo, Japan, 2005) and Hardy-Rand-Rittler pseudo-isochromatic plates (HRR; 4th edition 2002, Richmond Products, Albuquerque, NM). Rayleigh anomaloscopy was performed, as described elsewhere (Dees & Baraas, 2014; Pedersen et al., 2018), in the dominant eye of all red-green colour vision deficient males, the protan carrier and 34 of the normal trichromats (15 males) (HMC Oculus Anomaloscope MR, Typ 47700, Oculus Optikgeräte GmbH, Wetzlar, Germany).

Informed consent was obtained from all participants after explanation of possible consequences of the study and prior to the experiments. The research was approved by the Regional Committee for Medical Research Ethics for the Southern Norway Regional Health Authority and was conducted in accordance with the principles embodied in the Declaration of Helsinki.

### 2.2. Genetics

All participants gave saliva samples (Oragene-DNA, OG-500, DNA Self-Collection Kit, DNA Genotek Inc., Ottawa, ON, Canada) for genetic analysis of their cone opsin genes. DNA was extracted, the L and M cone opsin genes were amplified by polymerase chain reaction (PCR), and exon 2, 3 and 4 were sequenced by a 3500 Genetic Analyzer (Applied Biosystems, Foster City, CA, USA), as described previously (Dees, Gilson, Neitz, & Baraas, 2015). Single-nucleotide polymorphisms (SNP) genotyping by Sequenome MassArray (Sequenome Inc., San Diego, CA, USA) was used to analyse the opsin array composition (Davidoff, Neitz, & Neitz, 2016). The amino acids specified at spectral tuning sites were used to determine the peak sensitivities for the L and M cone opsins (Asenjo, Rim, & Oprian, 1994; Neitz & Neitz, 2011). The genetic analyses were performed in the Neitz Lab at University of Washington, Seattle.

### 2.3. ERG flicker photometry for estimating L:M cone ratios

Spectral sensitivity functions were measured in the dominant eye with full-field ERG flicker photometry at a temporal frequency of 31.25 Hz, using a method described elsewhere (Carroll et al., 2000; Jacobs, Neitz, & Krogh, 1996; McMahon, Carroll, Awua, Neitz, & Neitz, 2008), with a modified version of the instrument described by Carroll et al. (2000). The ERG signals were created by 4 LEDs (Swanson, Ueno, Smith, & Pokorny, 1987) and presented in Maxwellian view through a Meade 30 mm telescope lens. The ERG system was calibrated by measuring the LED wavelength emission profiles with a spectrophotometer (SpectraScan PR650, Photo Research, NY, USA). The intensity of a monochromatic test light was consecutively adjusted until the ERG signal exactly matched that produced by a fixed-intensity reference light (519 nm). The mean intensity from at least three independent measures for each of three test wavelengths (465 nm, 634 nm and 655 nm) was used for further analyses. Photopigment optical density (OD_L_ and OD_M_) was set to 0.35 and 0.22 for the L and M cone opsin, respectively (Carroll et al., 2000), and the data were corrected for lens absorption by an age-dependent lens correction (Pokorny, Smith, & Lutze, 1987). The spectral sensitivity data were then fitted with a weighted sum of individualized L and M cone spectral sensitivity functions, based on the genetically confirmed λ_max_ values for L and M, and estimated %L cones was calculated from the L and M weights [100 × L/(L + M)] (Carroll et al., 2000). The root mean squared error of the fit, on a scale from 0 to 1, was less than 0.05 in all participants included. The estimated cone ratios were adjusted by a factor of 1.5, as suggested by Hofer et al. (2005), to correct for the reported larger contribution of each M cone to the ERG signal when comparing with adaptive-optics imaging combined with retinal densitometry. One operator (author LAH) performed all ERG measurements. The test-retest reliability of the ERG system was measured by three independent measures of the L:M cone ratios performed on different days in two male and two female normal trichromats; see results in Table 1. The individual estimate of %L was never more than 6.1 % difference from the mean for the three measurements and showed a repeatability variation within ± 2.3% L cones. Cyclopentolate 1 % or Tropicamide 0.5% was administered to dilate the test eye prior to measurements. All ERG measurements were made in an illuminated room between 150 6and 300 lux.

In the estimate of the individual L:M cone ratio, the genetically confirmed L cone λ_max_ was used for all normal trichromatic males (*n* = 60; all had single L genes) and for all normal trichromatic females who had L cone opsin genes encoding spectrally *identical* L cone opsins in the two X-chromosomes (*n* = 33). A group of normal trichromatic females had L cone opsin genes in the two X-chromosomes encoding two L cone opsins with *distinct* λ_max_ (*n* = 43). Individual L:M cone ratios were estimated in three ways for these females: (1) based on mean λ_max_ for the two L cone opsins; (2) based on the L cone opsin with the highest λ_max_; and (3) based on the L cone opsin with the lowest λ_max_ These estimates define a range of potential L:M cone ratios for females with *distinct* L λ_max_, which is determined by the degree of X-chromosome inactivation in each cell (Jorgensen et al., 1992; Lyon, 1972; Sharp, Robinson, & Jacobs, 2000). Variation in M λ_max_ has been shown to have minimal impact on the estimated L:M cone ratio (Bieber, Kraft, & Werner, 1998). For the participants who had M cone opsin genes encoding spectrally *distinct* M cone opsins, mean M λ_max_ was used in the estimate of the individual L:M cone ratio.

### 2.4. Data analysis

Spherical equivalent refraction (SER) was calculated as sphere + 1/2 cylinder, wherein the sphere was defined as the most positive meridian of the autorefractor measurement in terms of a 13.5 mm vertex distance. Myopia was defined as SER ≤ −0.50D and hyperopia as SER ≥ +0.50D. Mean corneal radius (CR) was estimated as the mean of the corneal radii measured in the flattest and steepest meridians, and axial length (AL) was used to estimate AL/CR-ratios for each participant.

The analysis was performed by the statistical computing software R, version 3.4.0 (R Core Team, 2016). Correlations were assessed using Pearson (*r*_p_) coefficients, and linear regression analyses were performed with %L cones as the dependent outcome variable. Between-group differences were examined using one-way analysis of variance, and Student’s or Welch ‘s two independent sample *t* tests for equal or unequal variances, respectively. Pearson’s Chi-squared test and Fisher’s Exact test for count data were used to assess relationship between two categorical variables. Differences were considered significant when *p* ≤ 0.05. Datasets of all normal trichromats are available online (Hagen & Baraas, 2019).

## 3. Results

### 3.1. Estimated %L cones

**Fig. 1.**
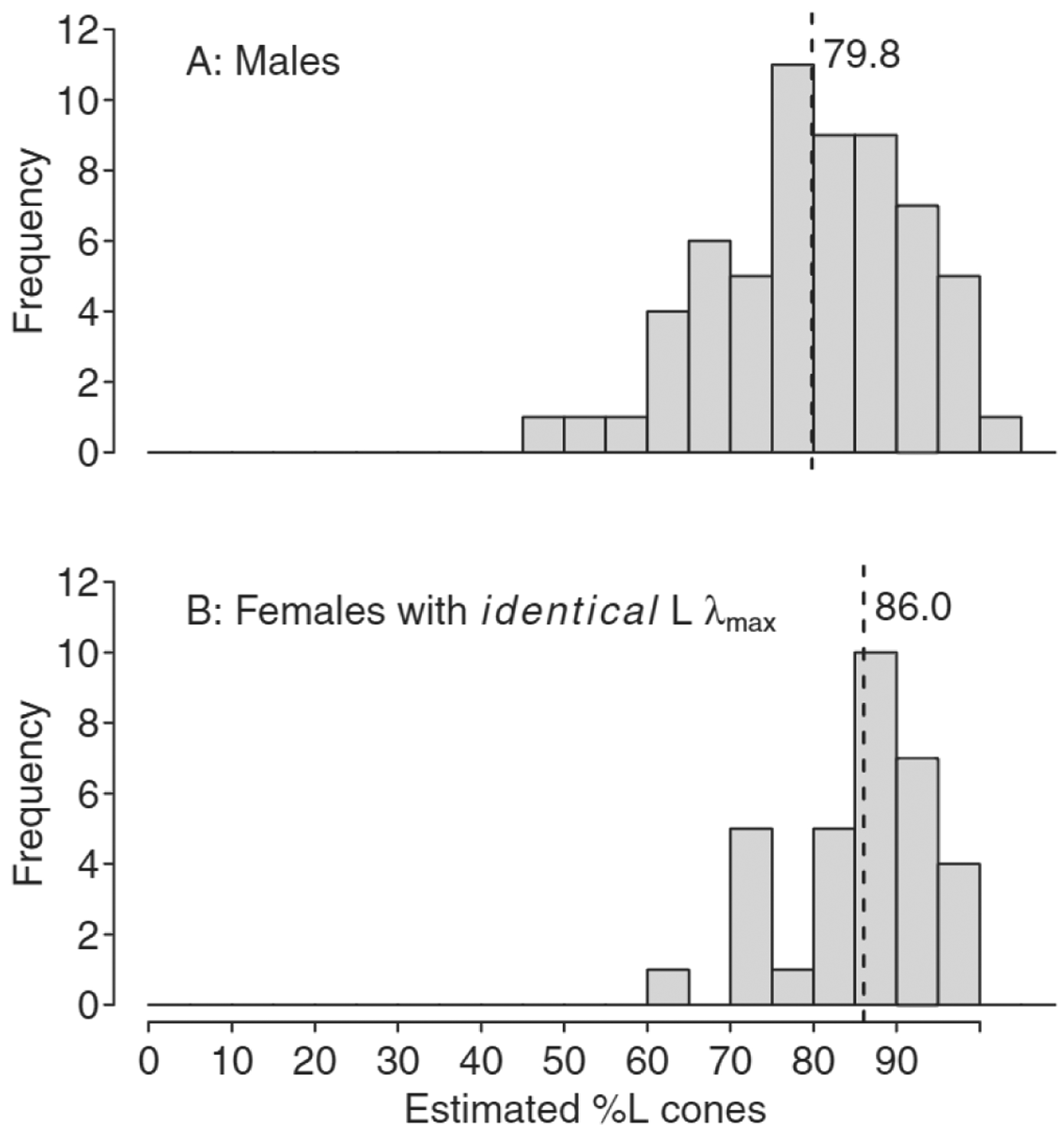
Distribution of estimated %L cones for (A) male normal trichromats (*n* = 60) and (B) female normal trichromats who had L opsin genes encoding *identical* L cone λ_max_ (*n* = 33). The dashed lines illustrate mean %L cones, which was significantly different between males and females [Mean (SD) 79.8 (± 11.8)% vs. 86.0 (± 8.6)%; *t*(91) = −2.66, *p* = 0.01]. Repeatability variation was estimated to ± 2.3% L cones.

**Fig. 2.**
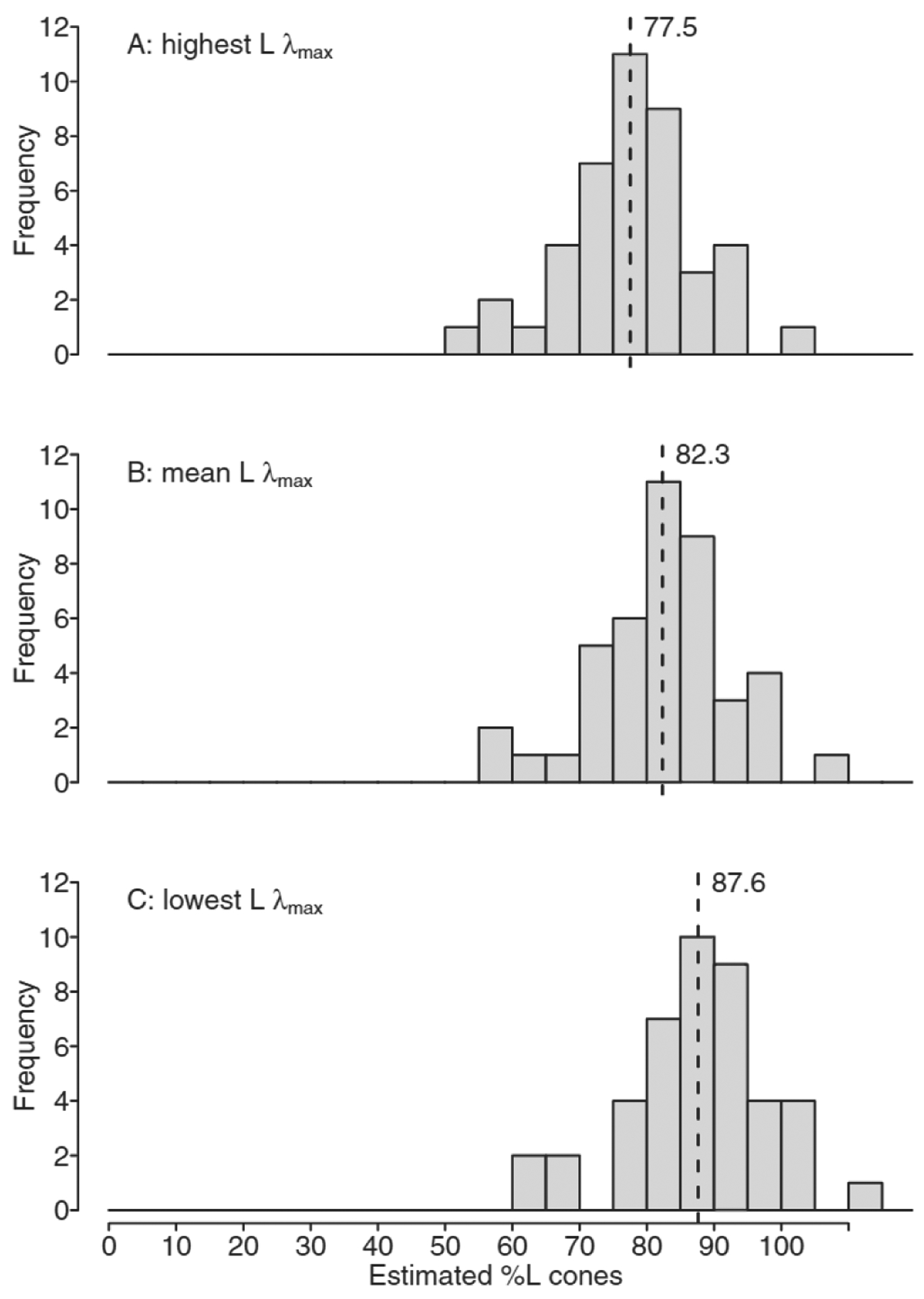
Distribution of estimated %L cones for female normal trichromats who had L opsin genes encoding *distinct* L cone λ_max_ (*n* = 43) based on (A) the individual L cone opsin with the highest λ_max_; (B) mean λ_max_ for their two L cone opsins; and (C) the individual L cone opsin with the lowest λ_max_ These estimates define a range of potential L:M cone ratios for females with *distinct* L λ_max_, which is determined by the degree of X-chromosome inactivation in each individual (Jorgensen et al., 1992; Sharp et al., 2000). The dashed lines illustrate mean %L cones.

Fig. 1 shows the distribution of estimated %L cones for male (Fig. 1A: *n* = 60) and female normal trichromats who had L opsin genes encoding *identical* L cone λ_max_ (Fig. 1B: *n* = 33). The estimated %L cones varied from 49.9% to 100.3% for the males and from 64.3% to 99.5% for females with *identical* L cone λ_max_ (all were within 100% L cones when considering a repeatability variation of ± 2.3% L cones). Females had a significantly higher mean (± SD) %L cones than males [86.0 (± 8.6)% vs. 79.8 (± 11.8)%; *t*(91) = − 2.66, *p* = 0.01]. Fig. 2 shows the distribution of estimated %L cones for the females who had L opsin genes encoding two *distinct* L cone λ_max_ (*n* = 43) based on the highest L λ_max_ for the two L opsins (Fig. 2A), the mean L λ_max_ (Fig. 2B) and the lowest L λ_max_ (Fig. 2C). The mean %L cones was 82.3 (± 10.0)%, with a possible range from mean 77.5% to mean 87.6%. Mean %L cones was also significantly higher for *all* females (*n* = 76; 83.9 (± 9.6)%) compared with males [*n* = 60; 79.8 (± 11.8)%; *t* (13 4) = −2.24, *p* = 0.03], when the mean L cone λ_max_ was used for those with *distinct* L λ_max_ under the assumption that each X-chromosome was silenced in half of the cells by X-chromosome inactivation for estimating mean %L cones.

### 3.2. Validation of ERG measurements and estimates of %L cones

Rayleigh match midpoint (MMP) correlated significantly with L λ_max_ for 15 male and 9 female normal trichromats with *identical* L λ_max_ (*r*_p_ = − 0.825, *p* < 0.001), as expected from previous studies (Winderickx et al., 1992), but not with estimated %L cones (*r*_p_ = 0.167, *p* = 0.44). Thus, the variation in L λ_max_ is removed as a source of error in the estimate of %L cones (Carroll et al., 2002). The results were the same when 10 females with *distinct* L λ_max_ were included in the analyses, with the estimate of %L cones based on their mean L cone λ_max_ (data for 34 normal trichromats: Rayleigh MMP versus mean L λ_max_: *r*_p_ = −0.825, *p* < 0.001; Rayleigh MMP versus %L cones: *r*_p_ = 0.068, *p* = 0.70). Mean (± SD) Rayleigh MMP and matching range (MR) for normal trichromats were MMP = 42.4 (± 2.1) and MR = 2.8 (± 1.3) for 15 males, MMP = 41.1 (± 2.0) and MR= 2.5 (± 1.9) for 9 females with identical L λ_max_, and MMP = 41.4 (± 1.2) and MR = 2.5 (± 1.4) for 10 females with distinct L λ_max_

Table 2 shows the Rayleigh match results and estimated %L cones for the red-green colour vision deficient male controls. The protan controls were estimated to have approximately 0% L cones. The estimated %L cones for the 13- and 66-year old deuteranope controls were 98% and 88%, respectively. The discrepancy from 100% L cones in the 66-years-old deuteranope, may be due to over-compensation for changes in ocular media with age. The clarity of his crystalline lenses was evaluated using the Lens Opacities Classification System III (LOCS III) (Chylack et al., 1993) and nuclear opalescence was graded and found to be lower than NO2. Nucleus staging was measured to grade 2 with Pentacam HR (Oculus, Typ 70900, Wetzlar, Germany), which is directly comparable with LOCS III NO grade (Pei, Bao, Chen, & Li, 2008). This implies that his lens density was more akin to someone aged 38 years (Pesudovs, Marsack, Donnelly, Thibos, & Applegate, 2004). Choosing a lens density for a 38-year-old gives an estimate of 101.2% L cones for the 66-year-old deuteranope. The deuteranomalous male control had a Rayleigh MMP as low as 16.2, which has been associated with a high OD_L_ (Thomas & Mollon, 2004). An increase of the OD_L_ in the estimate of %L from the fixed value of 0.35 to 0.55, results in a decrease in the estimate of %L cones from mean 107.2% (range: 102–112%) to mean 100.6% (96–105%) for the deuteranomalous male (given that he has two different L cone opsins with λ_max_ 553.0 and 555.5 nm). The genetically-confirmed protan carrier control had an estimate of 39% L cones, which was lower than any of the female normal trichromats. She was also heterozygous for the S-opsin mutation Tl 901, which causes abnormal S-cone function (Baraas, Hagen, Dees, & Neitz, 2012).

### 3. 3. Estimated %L cones related to S18OA and photopigment optical density

Table 3 shows the frequency of haplotypes encoded by exon 3 on the L cone opsin gene and the associated expected % correctly spliced transcripts (Buena-Atienza et al., 2016; Greenwald et al., 2017; Neitz & Neitz, 2018). Five dimorphic amino acid positions are specified by exon 3; L153M, V171I, A174V, I178V, and S180A (single letter amino acid codes are: L = leucine, M = methionine, V = valine, I = isoleucine, A= alanine, S = serine). Serine versus alanine at position 180 (S180A) is the only amino acid substitution encoded by exon 3 that shifts the spectral tuning of the opsin (Neitz & Neitz, 2011). Having serine versus alanine at L position 180 was not significant predictor for %L cones (*β* = −4.0, *p* = 0.07) when adjusted for sex in a linear regression [*F*(2, 90) = 5.30, *p* = 0.007, R^2^ = 0.11] in the group of males and females with *identical* L λ_max._ See Table 4 for frequency of L and M cone λ_max_.

Amino acid substitutions encoded by exon 2 have been suggested to regulate the optical density of the M cone opsin (Neitz, Neitz, He, & Shevell, 1999), whether this applies to the L cone opsin is not known. Increasing the OD_L_ from the fixed value decreases the estimated %L cones, while a change in OD_M_ has minimal effect on the estimated %L cones. Here, 131 of the normal trichromats (96.3%) had the exact same haplotypes encoded by exon 2 on the L cone opsin gene (TIS). Five females had a different L exon 2 haplotype that may be related to a different optical density of the L cone [one female with *identical* L cone λ_max_ (98.0% L), and 4 females with *distinct* L cone λ_max_ (73%, 78%, 81 % and 96% L based on mean L cone λ_max_)]. Removing these females from the group had no effect on the mean %L cones.

### 3.4. Comparison with other studies

Table 5 gives an overview of mean %L cones and the proportion of S180A from present and other studies. The mean %L cones for the Norwegian male normal trichromats [79.8 (± 11.8)% L] was significantly higher than that reported for African and African American (McMahon et al., 2008) [65.1 (± 10.7)% L; *t*(85) = 5.53, *p* < 0.001], American Caucasian (Carroll et al., 2002; Hofer et al., 2005) [73.1 (± 11.1)% L; *t*(120) = 3.24, *p* = 0.002], and East Asian (Kuchenbecker et al., 2014; Yamauchi et al., 2013) male normal trichromats. L:M cone ratios in all studies were measured by ERG flicker photometry. Table 5 shows that Caucasians (Deeb, Alvarez, Malkki, & Motulsky, 1995; Winderickx, Battisti, Hibiya, Motulsky, & Deeb, 1993) are reported to have a higher proportion of alanine at L position 180 than African (Deeb & Motulsky, 1996) and Japanese (55% versus 20%) (Deeb et al., 1995; Hayashi, Ueyama, Tanabe, Yamade, & Kani, 2001).

### 3.5. Refractive error

SER, astigmatism and axial length correlated between the right and the left eye (*n* = 136; SER: *r*_p_ = 0.94; refractive astigmatism: *r*_p_ = 0.43; axial length: *r*_p_ = 0.92; all *p* < 0.001). Thus, in further analysis, data from the right eye were used. Table 6 shows mean SER, ocular axial length (AL), corneal curvature (CR), and the frequency of refractive errors for the right eye of normal trichromats along with estimated %L cones for the groups.

**Fig. 3.**
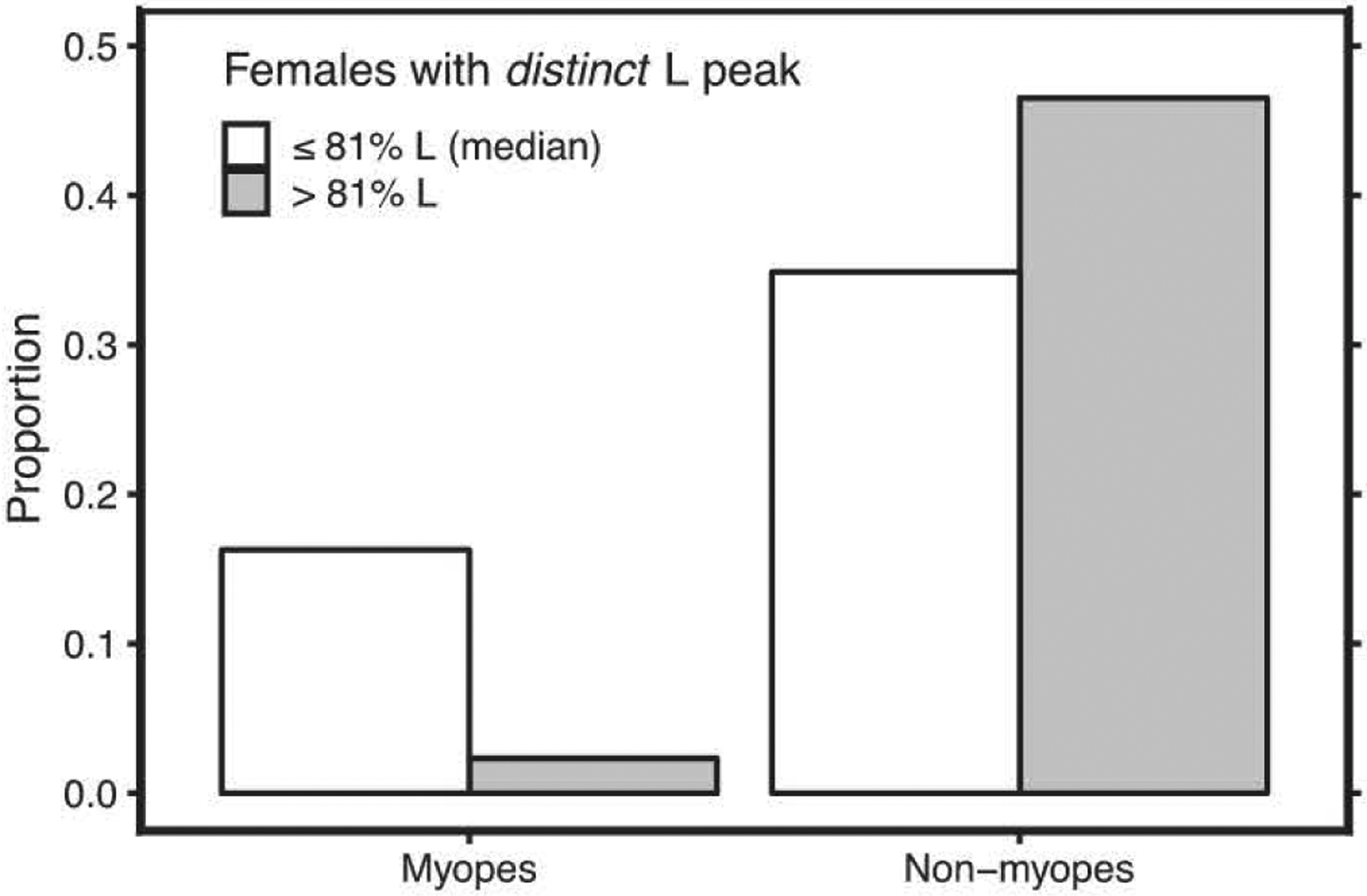
Association between %L cones and myopia in females with *distinct* L λ_max_ (*n* = 43; median ≤ 81 % L; Fisher’s exact test *p* = 0.05). The participants were grouped by %L (white bars: %L ≤ median; grey bars: %L > median).

Fig. 3 shows the proportion of myopic females with two *distinct* L cone λ_max_ who had low (%L ≤ median) or high %L cones (%L > median). When comparing myopes with non-myopes (emmetropes and hyperopes) in this group, myopia was found to be significantly more frequent in those with low vs. high %L cones (*n* = 22 vs. 21; 31.8% vs. 4.8% myopia; Fisher’s exact test *p* = 0.046). Those with low %L cones were also more myopic than those with high %L cones [*n* = 22 vs. 21; mean (SD) SER −0.07 (± 1.2)0 vs. 0.81 (± 0.7)0; Welch *t* (33.1) = −2.91, *p* = 0.006]. Likewise, in the group of *all* females (*n* = 76), mean SER was more myopic in those with low %L cones than in those with high %L cones [n = 39 vs. 37; −0.03 (± 1.2)D vs. 0.58 (± 0.8)D; Welch *t*(67.8) = −0.52, *p* = 0.01]. There were no associations between estimated %L cones or L and M cone opsin genetics and refractive error or ocular biometry for the males, but the number of male myopes was low (*n* = 5).

Table 7 shows that there was a significant association between the frequency of refractive error in females and whether a female was homozygous or heterozygous for their specific L exon 3 haplotype(s) (Pearson Chi-Squared test based on 9999 Monte-Carlo resamplings, *p* = 0.008), with less ametropia and more emmetropia among the females who were homozygous for their specific L exon 3 haplotype. Males are, by definition, never heterozygous.

## 4. Discussion

The results presented here are consistent with the hypothesis that the L:M cone ratio, combined with opsin gene polymorphism and exon 3 haplotypes with less severe splicing defects are implicated in susceptibility to myopia-generating environmental triggers (Neitz & Neitz, 2015; Zhou et al., 2015). The high myopia prevalence in East Asians (Lin, Shih, Hsiao, & Chen, 2004; Pan, Dirani, Cheng, Wong, & Saw, 2015) is not observed in Norwegians despite high educational pressure and low daily light exposure due to few daylight hours in the autumn-winter period (Hagen et al., 2018), but they have a significantly higher mean L:M cone ratio than that previously reported for East Asians (Kuchenbecker et al., 2014; Yamauchi et al., 2013). Furthermore, females with low %L cones (symmetric L:M cone ratios) were on average more myopic than females with high %L cones (skewed L:M cone ratios). Myopia prevalence was higher in females who were heterozygous for the L opsin exon 3 haplotype than in the homozygous females.

### 4.1. The association between cone opsin and myopia

It is well known that high-grade myopia is associated with rare interchange exon 3 haplotypes, such as LVAV A and LIAVA, of the L or M opsin genes (Carroll et al., 2012; Greenwald et al., 2017; McClements et al., 2013; Orosz et al., 2017) (none in our sample; Table 3), resulting in incorrect splicing of exon 3 and greatly reduced amount of functional opsin in the cones harbouring the mutation. Eye growth associated with rare interchange haplotypes is suggested to be caused by erroneous contrast signals produced by mosaics with both normal cones and cones with mutant opsins (Greenwald et al., 2017; Patterson et al., 2018). It is not unlikely that this mechanism also plays a role in common myopia, because there is large between-individual variation in the amino acid sequences of the L and M opsin genes. Amino acid substitutions can have a less deleterious effect on the cone opsin function than for example LVAVA and LIAVA, without altering the spectral sensitivity or λ_max_ (Carroll et al., 2002; Neitz et al., 1999). How effectively cone photoreceptors signal contrast and spatial frequency information depends on the gene code of opsins expressed in the L and M cones (Greenwald et al., 2017) as well as the organization and the ratio of L and M cones. The L/M gene array of colour normal males and homo-zygote females will give a cone mosaic expressing one type of L and one type of M opsin. The number of cones harbouring a less than normal functioning opsin depends on whether the opsin is L or M, whether the amino acid substitution resides on the first or second gene on the array, and the L:M cone ratio, resulting in differences in the ratio of less-than-normal to normal functioning cones. If less-than-normally functioning cones causes ON bipolar cells to signal more contrast than is actually present, then a high contrast spatial-frequency pattern, that moves across the retina due to eye movements, will give rise to less synchronized signals from the ON bipolar cells to ganglion cells and poorer signal fidelity (Ala-Laurila, Greschner, Chichilnisky, & Rieke, 2011). These errors in signalling of spatial contrast information could be the step that sets off the signalling cascade that stimulates eye growth (Wallman & Winawer, 2004). Another factor that may play a role is the organization of L and M cones in patches of the same cone type (Hofer et al., 2005). This patchiness is advantageous for signalling of achromatic spatial information of high spatial frequency (high contrast fine details), as neighbouring cones of different types will give rise to chromatic noise (undesired differences in spectral information) which degrades the achromatic spatial signal (Osorio & Vorobyev, 2008; Roorda, Metha, Lennie, & Williams, 2001; Williams, Sekiguchi, Haake, Brainard, & Packer, 1991). Skewed L:M cone ratio (near 0% or near 100% L cones) makes it more likely that neighbouring cones are of the same type, improving signalling of spatial information (if all cones are normally functioning with the same level of opsin expression). The most skewed L:M cone ratios are found in red-green colour vision deficient individuals, as they have only L *or* M cones in the retina (in addition to the more sparsely distributed S cones) leading to high resolution, low noise signalling. Common forms of congenital red-green colour vision deficiency are indeed associated with low myopia susceptibility and prevalence (Ostadimoghaddam et al., 2014; Qian et al., 2009). In Norway, 8% of males are red-green colour vision deficient, and about 15% females are assumed to be deutan or protan carriers (Baraas, 2008; Waaler, 1968). Higher L:M cone ratios are expected in females when samples of normal females include carriers of deutan colour vision deficiency who have higher L:M ratios than normal males and non-carrier females. The females with highly skewed cone ratios provide a sample within the Norwegian population in which the hypothesis that biased cones ratios protect against myopia can be tested.

### 4.2. Heterozygosity of common L opsin exon 3 haplotypes

That the L:M cone ratio combined with L opsin exon 3 haplotypes that give rise to mild splicing defects play a role in myopia susceptibility could also explain why myopia prevalence was the same in females who were homozygous for the L opsin exon 3 haplotype as in the males (9% and 8% respectively; Table 7), but much higher in females who were heterozygous for the L opsin exon 3 haplotype (24% myopia). Because females have two X chromosomes, L and/or M opsin exon 3 haplotype heterozygosity translates into a retina where there will be patches with two sets of L and/or M cones expressing different haplotypes, and these haplotypes could give rise to less-than-normally functioning opsin and/or altered spectral sensitivity. It has been shown that females with heterozygote mosaics will vary greatly in chromatic contrast sensitivity, depending on opsin haplotype (Dees et al., 2015) and their L:M ratio (Gunther & Dobkins, 2002). Those with haplotypes that code for more than two different L and/or M cones with large spectral separation and have a low, symmetrical L:M cone ratio, will have improved chromatic sensitivity (Osorio & Vorobyev, 1996), but increased chromatic noise degrading signalling of high-spatial frequency information (Barlow, 1982; Osorio, Ruderman, & Cronin, 1998). This suggests that the sex difference in myopia prevalence could be a consequence of heterozygosity of common L opsin exon 3 haplotypes.

### 4.3. Serine versus alanine at L opsin position 180 (S180A)

A common polymorphism on exon 3 of the L opsin that affects spectral separation between Land M cones is serine versus alanine at position 180 (Sl80A). Serine shifts the L cone λ_max_ 3–4 nm (Asenjo et al., 1994; Neitz, Neitz, & Jacobs, 1991), and is known to result in higher sensitivity to red than alanine (Winderickx et al., 1992). A significant green shift has been reported in myopes compared with emmetropes and hyperopes (Rucker & Kruger, 2006), as well as an association between a green shifted Rayleigh match and increased myopia (Wienke, 1960). It is plausible to assume that the myopes in these reports likely had serine, since green shifted (lower) Rayleigh match midpoints are a signature of serine at position 180 (Winderickx et al., 1992). The proportion of Sl80 was significantly lower in the Norwegian male normal trichromats (45% have serine) than that reported for East Asians (80% have serine) and other more southerly located populations with almost no seasonal variation in daylight (Deeb et al., 1995; Hayashi et al., 2001) (Table 5). But why would a population living at northern latitudes evolve an eye that also is protective against developing myopia? Studies of cone opsin genes in primates indicate that having alanine at position 180 in the L opsin may be an evolutionary result of adaptation to long periods of low light levels (Jacobs, 2008; Jacobs et al., 2017). X-linked cone opsin variations across a lemur clade shows that the most strictly diurnal lemur has serine at position 180, whereas the lemur that is generally diurnal, but also is active at dusk/dawn, has alanine at position 180 (Jacobs et al., 2017). The platyrrhine Aotus, the only anthropoid (monkey) considered to be nocturnal, is reported to only have alanine (Jacobs, 2008). The decrease of L cone λ_max_ and narrowing of the separation between L and M cone λ_max_, as a consequence of alanine at position 180, not only improves signal-to-noise ratio when the light is bright, as mentioned in 4.1. above, but also when the light is dim, as it reduces dark noise (Lewis & Zhaoping, 2006). This may be an advantage if you spend many hours indoors in low light levels doing near work (Mountjoy et al., 2018; Wu et al., 2018).

### 4.4. Possible limitations

A larger sample size could have strengthened the results. The low number of myopes reflects the low myopia susceptibility in this population. Further work is needed to see if these findings can be duplicated in a population with high myopia susceptibility.

### 4.5. Conclusions

High L:M cone ratios are previously suggested to protect against myopia development (Neitz & Neitz, 2015; Zhou et al., 2015), and the results here, showing that Norwegians have higher mean %L cones than East Asians, and that Norwegian females with high %L cones were less myopic, support this theory. Any advantage associated with photoreceptor function during dim light will necessarily also be related to the role circadian clocks play in modulating photoreceptor electrical coupling during day and night and in anticipation of changing light levels (Felder-Schmittbuhl et al., 2018).

## Figures and Tables

**Table 1:** Repeatability measurements of the ERG’s %L estimates. Individual estimate of % L from three independent ERG measurements on three different days for four normal trichromats; two males (both with single L genes) and two females (one with *identical* L λ_max_ and one with *distinct* L cone opsin λ_max_). Individual %L estimates were compared with the mean for the three measurements (|%L – mean|). Mean difference of individual estimates from the mean was 2.3%.

Measure no.	Male A: Single L gene		Male B: Single L gene		Female A: *Identical* L λ_max_		Female B: *Distinct* L λ_max_	
	%L	|%L – mean|	%L	|%L – mean|	%L	|%L – mean|	%L	|%L – mean|
1	52.9	2.1	55.7	2.1	62.9	0.8	57.7	4.0
2	50.0	0.8	58.9	1.2	61.1	2.6	59.5	2.1
3	49.6	1.2	58.7	0.9	67.0	3.3	67.8	6.1
Mean	50.9	1.4	57.8	1.4	63.7	2.2	61.7	4.1

**Table 2:** Rayleigh match results and estimated %L cones for the five males who served as controls for validation of ERG measurements and estimates of %L cones, who had known cone opsin genes conferring red-green colour vision deficiency.

Color vision deficiency	Age [years]	Opsin array	λ_max_ [nm]	Rayleigh match		Estimated %L cones	Adjusted %L cones*
				MMP	MR		
Protanope	22	M	530	36.5	73.0	−0.8	-
Protanomalous	21	MMM	533/530	68.0	3.8	−0.3	-
Deuteranope	66	L	559	36.4	72.7	87.6	101.2 (age: 38)
Deuteranope	13	L	559	36.2	72.4	98.4	-
Deuteranomalous	16	LL	555.5/553	16.2	10.0	102.4–111.9 (OD_L_ = 0.35)	96.1–105.1 (OD_L_ = 0.55)

* Adjusted %L cones are for the 66-year-old deuteranope when lens density was set to estimated age based on measured nuclear opalescence grade, and for the deuteranomalous based on a higher OD_L_, See main text for details.

**Table 3:** Frequency (%) of haplotypes encoded by exon 3 on the L cone opsin gene and the associated expected % correctly spliced transcripts (Buena-Atienza et al., 2016; Greenwald et al., 2017; Neitz & Neitz, 2018) for male normal trichromats (*n* = 60) with one L cone opsin gene and females (*n* = 33) who had L opsin genes encoding *identical* L cone λ_max_ and had *identical* L exon 3 haplotypes in their two L cone opsin genes.

Exon 3 L cone opsin gene*	Expected % correctly spliced transcripts	Males (*n* = 60)	Females *Identical* L λ_max_ (*n* = 33)
LVAIA	> 75	26.7	15.2
MVAIA	> 75	21.7	18.2
MVVVA	>75	3.3	0.0
LVVIA	> 75	1.7	0.0
MVVIA	> 75	1.7	0.0
LVAIS	100	28.3	21.2
LIAIS	100	6.7	0.0
MVAIS	100	5.0	6.0
MVVIS	100	3.3	0.0
LVVIS	100	1.7	0.0
Multiple		0.0	39.4

* Five dimorphic amino acid positions are specified by exon 3; L153M, Vl71I, Al74V, I178V, and S180A. The single letter amino acid code used here is as follows: L = leucine, M = methionine, V = valine, I = isoleucine A = alanine, S = serine.

**Table 4:** Frequency (%) of L and M cone λ_max_ [nm] in Caucasian normal trichromats, grouped by sex and whether females have L opsin genes encoding *identical* or *distinct* L cone λ_max_. For those who have opsin genes encoding *distinct* L or M cone λ_max_, two values for λ_max_ are given.

		Males (*n* = 60)			Females *Identical* L λ_max_ (*n* = 33)		Females *Distinct* L λ_max_ (*n* = 43)			
M λ_max_		530	533	530/533	530	530/533	M λ_max_		530	530/533
L λ_max_	559	33.3	8.3	1.7	42.4	9.1	L λ_max_	555.5/559	74.4	9.3
	555.5	53.3	0.0	1.7	45.5	0.0		555/559	4.7	2.3
	555	1.7	0.0	0.0	0.0	0.0		556.5/559	2.3	4.7
	553	0.0	0.0	0.0	3.0	0.0		553/555.5	0.0	2.3

**Table 5:** An overview of the proportion of Sl80A in present and previous studies. Mean %L cones for all, and grouped by Sl80A, are presented for studies that have reported L:M cone ratios. N/A = not available.

		All	Serine at L opsin position 180		Alanine at L opsin position 180		Mean (SD) % L cones		
Ethnicity	Study	*n*	*n*	%	*n*	%	All	Serine_180_	Alanine_180_
Caucasian Norwegian colour normal males	Present study for males whom we have measured %L	60	27	45.0	33	55.0	79.8 (11.8)	77.3 (12.2)	81.9 (11.2)
Caucasian American colour normal males	Carroll et al. (2002) & Hofer et al. (2005)	62	35	56.5	27	43.5	73.1 (11.1)	75.4 (10.8)	70.2 (11.0)
Caucasian colour normal males	Winderickx et al. (1993)	75	46	62.2	28	37.8	N/A	N/A	N/A
African and African American colour normal males	McMahon et al. (2008)	27	26*	96.3*	1*	3.7*	65.1 (10.7)	64.4 (10.2)	84.5
Japanese males	Deeb et al. (1995)	49	41	83.7	8	16.3	N/A	N/A	N/A
Japanese colour normal males	Hayashi et al. (2001)	119	94	79.0	25	21.0	N/A	N/A	N/A

* Frequency are based on L λ_max_ (559 nm for serine and 555.5 nm for alanine).

**Table 6:** Mean (SD) SER, ocular axial length (AL), corneal curvature (CR), proportion of refractive errors, and mean (SD) estimated %L cones for the 136 normal trichromatic participants grouped by refractive error (MYO = myopia, EMM = emmetropia, HYP = hyperopia).

							Refractive error (%)			Estimated %L			
	*n*	Age	SER [D]	Range SER [D]	AL [mm]	CR [mm]	MYO	EMM	HYP	All	MYO	EMM	HYP
Males	60	16.8 (0.9)	+ 0.60 (0.9)	−2.32 to + 4.23	23.7 (0.7)	7.9 (0.3)	8.3	33.3	58.3	79.8 (11.8) *n* = 60	84.9 (7.7) *n* = 5	78.6 (13.4) *n* = 20	79.8 (11.4) *n* = 35
Females with *identical* L λ_max_	33	16.7 (0.9)	+ 0.15 (l.1)	−3.36 to + 1.83	23.4 (0.8)	7.8 (0.2)	21.2	39.4	39.4	86.0 (8.6) *n* = 33	84.9 (9.1) *n =* 7	88.0 (7.3) *n =* 13	84.7 (9.8) *n =* 13
Females with *distinct* L λ_max_	43	17.1 (1.0)	+ 0.36 (1.1)	−2.56 to + 2.97	23.3 (0.7)	7.7 (0.2)	18.6	20.9	60.5	82.3 (10.0) *n* = 43	77.4 (8.1) *n* = 8	83.8 (14.4) *n* = 9	83.3 (8.6) *n =* 26
All females	76	16.9 (1.0)	+ 0.27 (1.1)	−3.36 to + 2.97	23.3 (0.8)	7.8 (0.2)	19.7	28.9	51.3	83.9 (9.6) *n =* 76	80.9 (9.1) *n =* 15	86.3 (10.7) *n =* 22	83.8 (8.9) *n* = 39

**Table 7:** Frequency (%) of refractive errors in males (*n* = 60), *all* females (*n* = 76), and in all females grouped according to being homozygous (*n* = 22) or heterozygous (*n* = 54) for their specific L exon 3 haplotype(s). There was a significant association between the refractive error and homozygosity versus heterozygosity for the females (Pearson Chi-Squared test based on 9999 Monte-Carlo resamplings, *p* = 0.008).

			*All* females grouped by their L exon 3 haplotype(s)	
	Males	*All* females	Homozygous females	Heterozygous females
*n*	60	76	22	54
Myopia (%)	8.3	19.7	9.1	24.1
Emmetropia (%)	33.3	28.9	54.5	18.5
Hyperopia (%)	58.3	51.3	36.4	57.4
